# Classification of Muscle-Invasive Bladder Cancer Based on Immunogenomic Profiling

**DOI:** 10.3389/fonc.2020.01429

**Published:** 2020-08-18

**Authors:** Xianghong Zhou, Shi Qiu, Ling Nie, Di Jin, Kun Jin, Xiaonan Zheng, Lu Yang, Qiang Wei

**Affiliations:** ^1^Department of Urology, Institute of Urology, National Clinical Research Center for Geriatrics and Center of Biomedical Big Data, West China Hospital of Sichuan University, Chengdu, China; ^2^Department of Pathology, West China Hospital of Sichuan University, Chengdu, China

**Keywords:** muscle-invasive bladder cancer, tumor immunity, tumor microenvironment, the cancer genome atlas, bioinformatics

## Abstract

There is a significant heterogeneity in the immunotherapeutic responsiveness of each muscle-invasive bladder cancer (MIBC) patient. In our research, we aimed to identify a novel classification of MIBC based on immunogenomic profiling that may facilitate the reasonable stratification of prognosis and response to immunotherapy. The single-sample gene-set enrichment analysis (ssGSEA) was used to analyze the RNA-seq data of 29 important immune signatures from TCGA. Unsupervised hierarchical clustering was performed to identify an immunogenomic classification of MIBC. Then, we assessed the features of the classification in prognosis, immune infiltration, tumor-infiltration lymphocytes, HLA genes, and PD-L1 expression level. A total of 399 MIBC samples were included and three subtypes named Immunity_High, Immunity_Medium, and Immunity_Low were identified. The Immunity_High had a significant advantage in overall survival over the Immunity_Medium and Immunity_Low (*p* = 0.046 and *p* = 0.024). From Immunity_Low to Immunity_High, immune cell infiltration and stromal content showed an upward trend (*p* < 0.001). Meanwhile, Immunity_High was associated with a significantly higher proportion of TILs including dendritic cells resting, macrophages M1, mast cells resting, T cells CD4 memory activated, and T cells CD8^+^. And the expression levels of all HLA genes and PD-L1 of Immunity_High were the highest, consistent (*p* < 0.001). Two hundred ninety eight MIBC patients treated with immunotherapy from the IMvigor210 were included to form an independent validation cohort to verify the robustness of immunogenomic classification and the ability to predict the response to immunotherapy. This classification had potential clinical implications for predicting prognosis and immunotherapeutic responsiveness of MIBC patients.

## Introduction

Bladder cancer (BC) is one of the most common malignant tumors of the urinary system, and ~25% of BCs invade bladder muscles to become muscle-invasive bladder cancer (MIBC) (1). Patients with MIBC usually have a higher probability of recurrence and a worse survival prognosis (2). In the past 30 years, cisplatin-based combination chemotherapy has become the standard therapeutic option in unresectable, advanced, and metastasis MIBC with median overall survival (OS) of 14–15 months (3). However, due to the toxicity of the drug, more than 50% of patients do not meet the criteria for using cisplatin and mainly use carboplatin-based chemotherapy instead. In this case, the prognosis of these patients often decreases to <12 months (4, 5). Therefore, more effective and safe therapeutic options of MIBC are urgently needed.

Recently, new immunotherapy designed to inhabit several immune checkpoints, such as programmed cell death 1 receptor (PD-1), PD-ligand-1 (PD-L1), and cytotoxic T lymphocyte-associated protein 4 (CTLA-4), have been applied in a variety of tumors including MIBC and got some encouraging results (6). Since 2016, five immune checkpoint inhibitors (ICIs) for MIBC (including atezolizumab, pembrolizumab, nivolumab, durvalumab, and avelumab) have already received approval from the US Food and Drug Administration (US-FDA) for their significant advantages in prolonging patients' survival time (7, 8). However, immunotherapy is not equally effective for all patients. Several recent large clinical studies have demonstrated that only about 20% of patients can benefit significantly from immunotherapy (9, 10). Given the low response rate and high treatment costs of immunotherapy, we need to develop accurate classification methods to identify patients most suitable for immunotherapy.

Nowadays, advances in next-generation sequencing technology have indicated the relevance of the tumor immune microenvironment in terms of tumor behavior and may predict the response to ICIs more comprehensively (11–13). Several recent studies on BC have reported that enrichment in immune cells infiltration of BC tissue was a strong indicator for immunotherapy response (14, 15). Furtherly, the relative proportions of different types of T cells, macrophages, and other tumor-infiltration lymphocytes (TILs) are closely related to the immune characteristics and prognosis of BC and other tumors (16, 17). Besides, the significant correlation between the expressions of the PD-L1 and human leukocyte antigen (HLA) genes and the immunotherapy response has also been demonstrated by previous studies (8, 18, 19). Meanwhile, classification based on a single biomarker (such as PD-L1 expression) is insufficient to describe disease-related immune characteristics and interactions between important cell types comprehensively. A classification method capable of integrating these several immune markers is still an emerging need. Therefore, in order to find a new and promising classification method for disease-related immune characteristics and immunotherapeutic responsiveness, we tried to apply the single-sample gene-set enrichment analysis (ssGSEA) of the transcriptome data of 29 immune gene sets and identify a new classification of MIBC.

## Materials and Methods

### Data Collection and Processing

This study was based on two cohorts from two independent datasets of BC. The first cohort was the discovery cohort. The gene expression quantification data and clinical data of discovery were downloaded from The Cancer Genome Atlas Urothelial Bladder Carcinoma (TCGA-BLCA) (https://portal.gdc.cancer.gov/) on Jan 19, 2020. From the TCGA-BLCA cohort, we excluded patients who were with non-MIBC (T0 or T1), without complete follow-up records or without complete RNA-seq data. A total of 399 patients were included in the discovery cohort. In order to screen out the matrix data of mRNAs with gene properties, the gene expression profiles were compared with the human genome annotation GTF file, which was downloaded from the GenCode platform (https://www.gencodegenes.org/). Then, the matrix data of the gene expression values were processed by the Perl software.

The second cohort was the validation cohort. The gene expression quantification data and clinical data were obtained from the supplementary data of IMvigor210. IMvigor210 cohort included patients with metastatic urothelial cancer who were treated with immunotherapy (atezolizumab, an anti-PD-L1 agent) (20). We excluded all patients who lacked immunotherapy response information and follow-up information. A total of 298 patients were included in the validation cohort. The validation cohort was used to verify the robustness of the classification of muscle-invasive bladder cancer based on immunogenomic profiling and whether it can distinguish patients with different responses to immunotherapy. The processing of the validation cohort was based on IMvigor210CoreBiologies, a package for the R statistical computing environment.

The detailed clinicopathologic characteristics of the study population are provided in the Supplementary Tables 1, 2.

### Clustering of MIBC Samples

In the discovery cohort, ssGSEA was used to quantify the enrichment levels of 29 immune signatures in each MIBC sample in the form of ssGSEA scores. We obtained the signatures for immune cell types from previous publications (21–23). (Supplementary Table 3) With this method, we could calculate the relative abundance of 29 immune gene sets in each tumor sample. Then, we performed unsupervised hierarchical clustering (complete linkage method) of MIBC based on the ssGSEA scores of the 29 immune signatures to establish an immunogenomic classification of MIBC.

After clustering and distinguishing subtypes of MIBC based on immunogenomic profiling, we studied the characteristics and verified the differences for the several MIBC immunogenomic subtypes from multiple aspects of tumor molecular features and phenotypes.

### Survival Analyses

We compared the overall survival (OS) of MIBC patients in different immunogenomic subtypes. The log-rank test was performed to test if the differences in survival time were significant. Kaplan-Meier curves were used to visualize the survival of different subtypes and the differences among them. We also performed a univariate Cox regression analysis to determine the hazard ratio (HR) of each subtype.

### Evaluating the Correlation of MIBC Immunogenomic Subtypes With Molecular Features

First, by the “Estimation of STromal and Immune cells in MAlignant Tumor tissues using Expression data” (ESTIMATE) method, the level of immune cell infiltration, stromal content, and tumor purity of each MIBC sample were calculated in the form of different scores (24). Then, we compared the scores of immune cell infiltration, stromal content, and tumor purity among different MIBC immunogenomic subtypes to reveal the relationships between MIBC immunogenomic classification and immune infiltration.

Furtherly, tumor-infiltrating immune cells (TILs) in MIBC samples were assessed by applying the “Cell type Identification By Estimating Relative Subsets Of RNA Transcripts CIBERSOFT” (CIBERSORT) deconvolution algorithm (25). CIBERSORT was used to analyze the relative expression levels of 547 genes in samples according to their gene expression profiles, to predict the proportion of 22 types of TILs in each sample (25). The gene expression signature matrix of 22 tumor-infiltrating immune cells was obtained from the CIBERSORT platform (https://cibersortx.stanford.edu/). We set 100 permutations and *P* < 0.05 as the criteria for the successful deconvolution of a sample. Then, we compared the proportions of the immune cell subsets between MIBC immunogenomic subtypes using the Mann–Whitney *U*-test.

Then, we calculated the expression levels of the HLA genes and PD-L1 in each subgroup. The differences in expression levels of HLA genes and PD-L1 among MIBC immunogenomic subtypes subgroups were assessed by Kolmogorov-Smirnov test.

### Independent Validation of the Existence of Immunogenomic Subtypes

In the validation cohort, in order to verify the robustness of this classification, we calculated the ssGSEA scores of the 29 immune signatures for each patient and performed unsupervised hierarchical clustering to identify the immunogenomic subtypes again. Then, we compared the immune cell infiltration, stromal content, and tumor purity among different immunogenomic subtypes from the validation cohort by the ESTIMATE algorithm.

### Evaluating of the Association of Immunotherapeutic Responsiveness With Immunogenomic Subtypes

In the validation cohort, all the patients had been treated with atezolizumab, an anti-PD-L1 agent. The patient's response to immunotherapy was divided into four situations: Complete Response (CR), Partial Response (PR), Stable Disease (SD), Progressive Disease (PD). We compared the response to immunotherapy among different immunogenomic subtypes to verify whether the immunogenomic subtypes have the potential to predict the immunotherapeutic responsiveness. Furtherly, we used Kaplan–meier method to analyze the OS of patients treated with immunotherapy in different immunogenomic subtypes.

### Statistical Analysis

The statistical software R (version 3.6.2), Perl (version 5.24.3), and EmpowerStats (version 2.0) were used in the above analyses. A *p* < 0.05 was considered statistically significant.

## Results

### Unsupervised Cluster Analysis Identified Three Immunogenomic Subtypes in MIBC Samples

**Figure 1:**
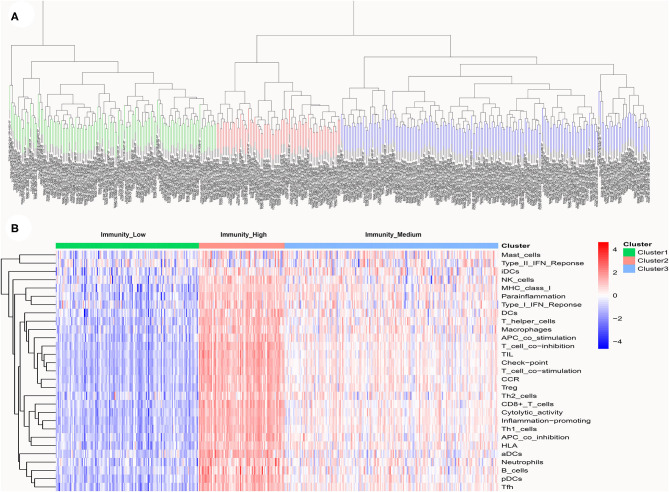
**(A)** Unsupervised hierarchical clustering of 399 muscle-invasive bladder cancer in the discovery cohort. **(B)** Enrichment of 29 immune gene sets in the three clusters yielded by the hierarchical clustering. We named the three clusters Immunity_High, Immunity_Medium, and Immunity_Low.

A total of 399 MIBC samples from TCGA were included in the current analyses. Based on the 29 immune-associated gene sets reported before which represented diverse immune cell types, functions, and pathways, we used the ssGSEA scores to quantify the activity or enrichment levels of immune cells, functions, or pathways in the cancer samples (21–23). Unsupervised hierarchical clustering analysis was then conducted to clearly divide the 399 MIBC samples into three clusters (Figure 1A). According to the heatmap of expression levels of the 29 immune-associated gene sets, we define the three clusters as three MIBC immunogenic subtypes named Immunity_High, Immunity_Medium, and Immunity_Low (Figure 1B). The specific score and immune immunogenomic subtype of each sample were shown in Supplementary Table 4.

### Correlation of MIBC Immunogenomic Subtypes With Prognosis

**Figure 2:**
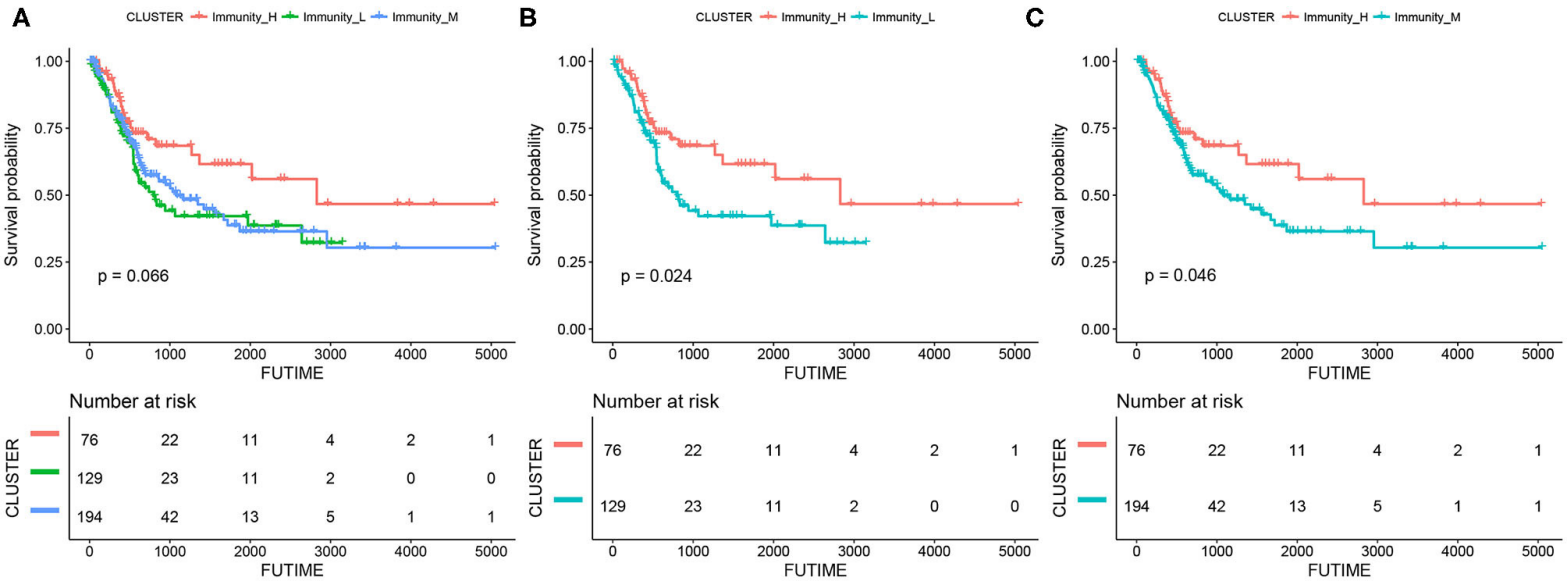
Kaplan-meier curves for overall survival (OS) of the three MIBC subtypes, including Immunity_High vs. Immunity_Medium vs. Immunity_Low **(A)**, Immunity_High vs. Immunity_Low **(B)**, and Immunity_High vs. Immunity_Medium **(C)**.

The results of survival analyses showed that the MIBC immunogenic subtypes had distinct clinical outcomes. In the Kaplan–meier analysis, the Immunity_High subtype had a significant advantage in OS over the Immunity_Medium (*p* = 0.046) and Immunity_Low subtypes (*p* = 0.024), while the difference in OS between the Immunity_Medium and the Immunity_Low subtypes was not significant (Figure 2).

### Correlation of MIBC Immunogenomic Subtypes With Tumor Microenvironment

**Figure 3:**
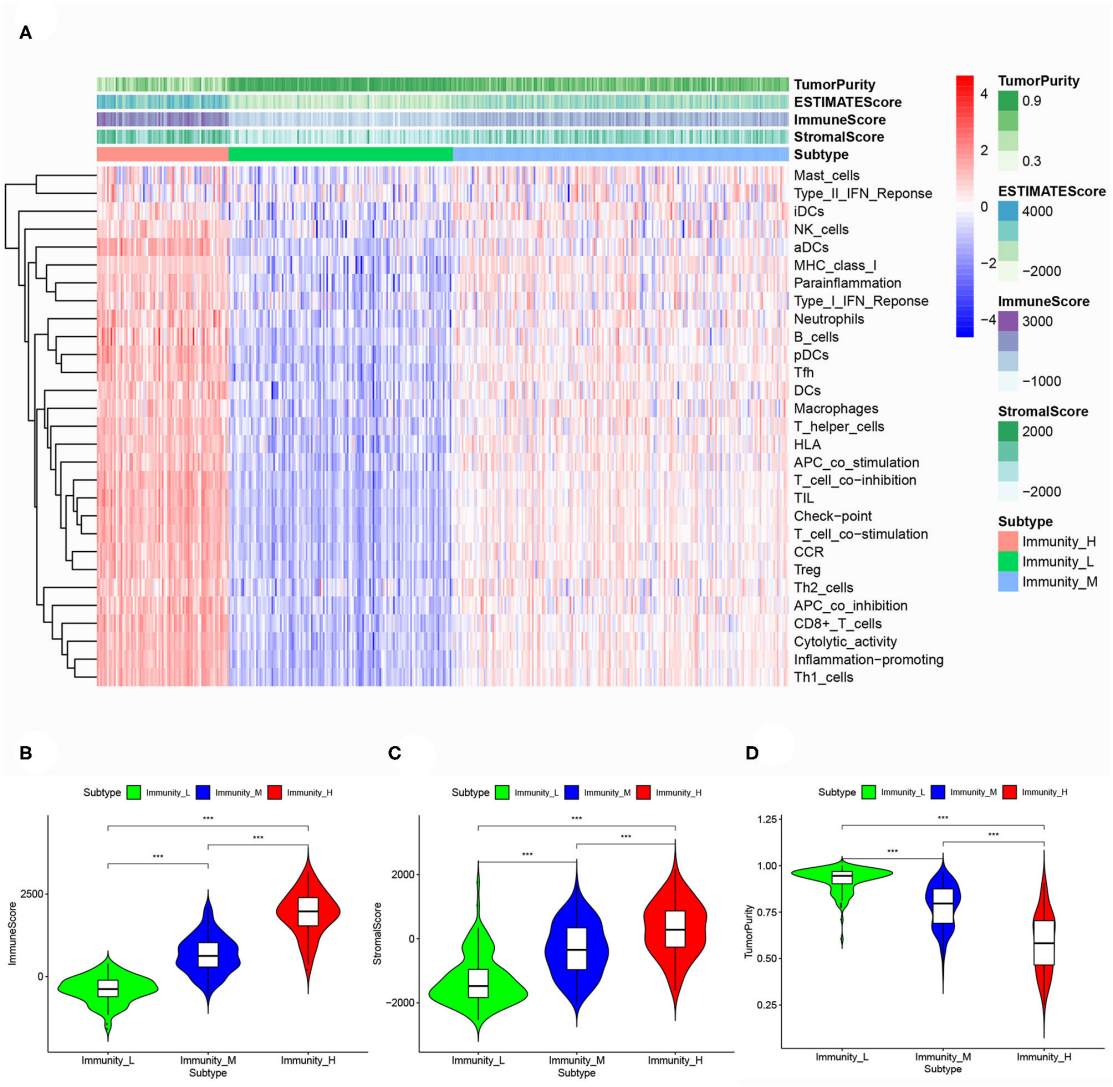
**(A)** The correlation between immunogenomic classification and the level of tumor immune infiltration was showed based immune score, stromal score, tumor purity score. The three types of scores were calculated by the ESTIMATE algorithm. The violin plots specifically showed the differences in the three subtypes in terms of immune cell infiltration level **(B)**, stromal cell content **(C)**, and tumor purity **(D)**. *p < 0.05, **p < 0.01, ***p < 0.001.

Based on the ESTIMATE algorithm, we successfully obtained the scores of immune cell infiltration, stromal content, and tumor purity. After combining the three types of scores with the expression heatmap of the 29 immune-associated gene sets, we could get some interesting findings. From Immunity_Low to Immunity_High, immune cell infiltration and stromal content scores showed an upward trend, indicating that the Immunity_High had a higher level of immune cell infiltration and stromal content. The changing trend of tumor purity was just the opposite. According to the order of Immunity_Low, Immunity_Medium, and Immunity_High, the tumor purity gradually increased (Figure 3A). The Kruskal–Wallis test confirmed that the differences among the three subtypes were all very significant in immune cell infiltration, stromal content, and tumor purity (*p* < 0.001; Figures 3B–D).

**Figure 4:**
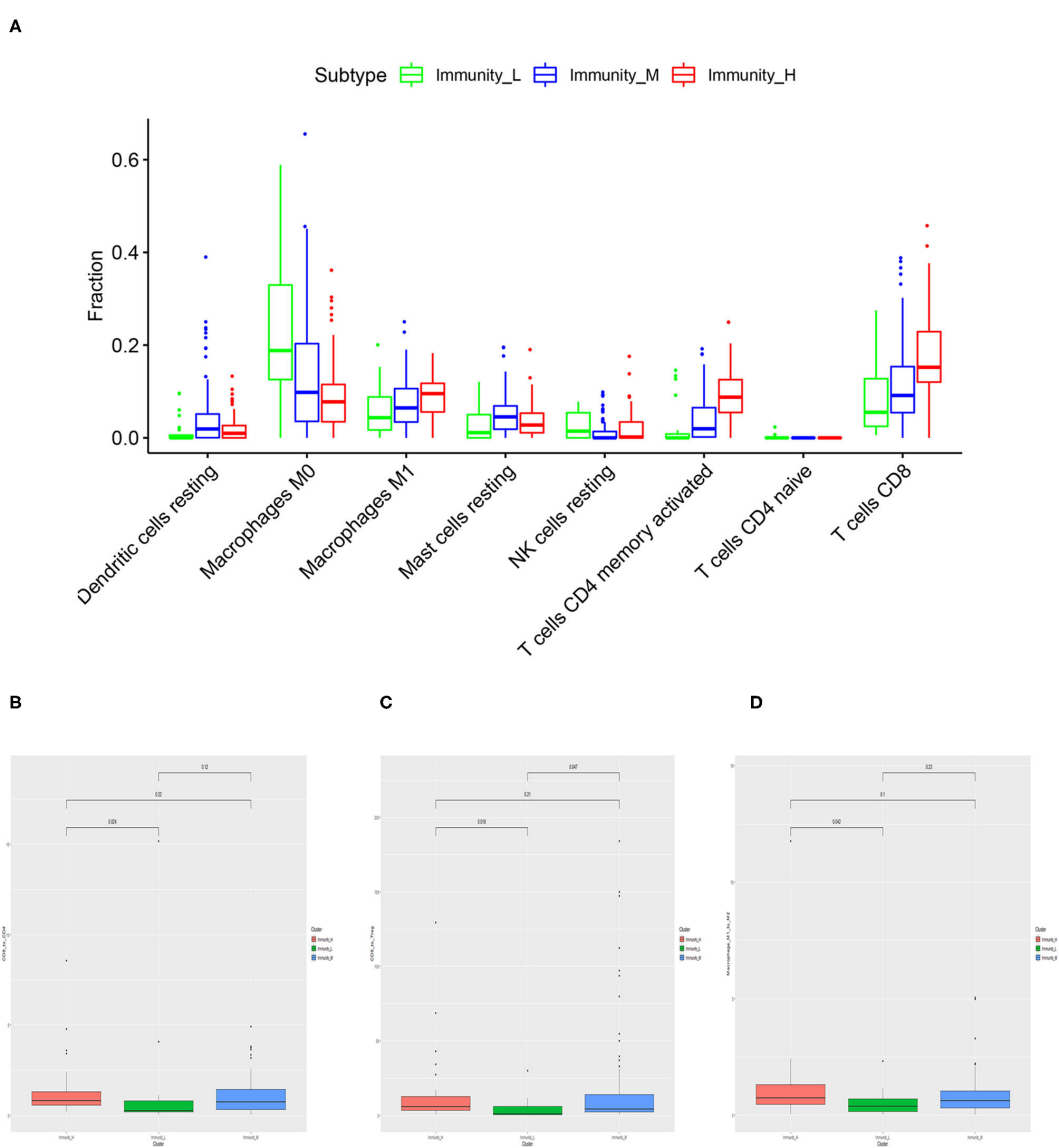
Specific tumor-infiltration lymphocytes with significantly different proportions among the three MIBC subtypes based on the CIBERSORT algorithm **(A)**. The differences of the ratio of T cells CD8 to T cells CD4 **(B)**, T cells CD8 to Treg **(C)**, and Macrophage M1 to M2 **(D)** among three MIBC subtypes.

Based on CIBERSORT algorithm, we systematically estimated the proportions of various TILs in MIBC samples (Figure 4A). The results showed the TILs subsets with significantly different proportions in different MIBC immunogenomic subtypes. Compared with Immunity_Low subtype, Immunity_High subtype was associated significantly with higher levels of TILs including dendritic cells resting, Macrophages M1, Mast cells resting, T cells CD4 memory activated, T cells CD8 (*p* < 0.01). We also found that higher proportions of Macrophages M0, NK cells resting, T cells CD4 naive were significantly associated with Immunity_Low (*p* < 0.001). Besides, we also explored several ratios between specific TIL like T cells CD8 to T cells CD4, T cells CD8 to Treg, and Macrophage M1 to M2 (Figures 4B–D). The results showed that patients in Immunity_H subtype had higher all three ratios significantly higher than those in Immunity_L subtype (*p* = 0.024, *p* = 0.018, *p* = 0.042; Figure 4B).

### Correlation of MIBC Immunogenomic Subtypes With HLA Genes and PD-L1 Gene

**Figure 5:**
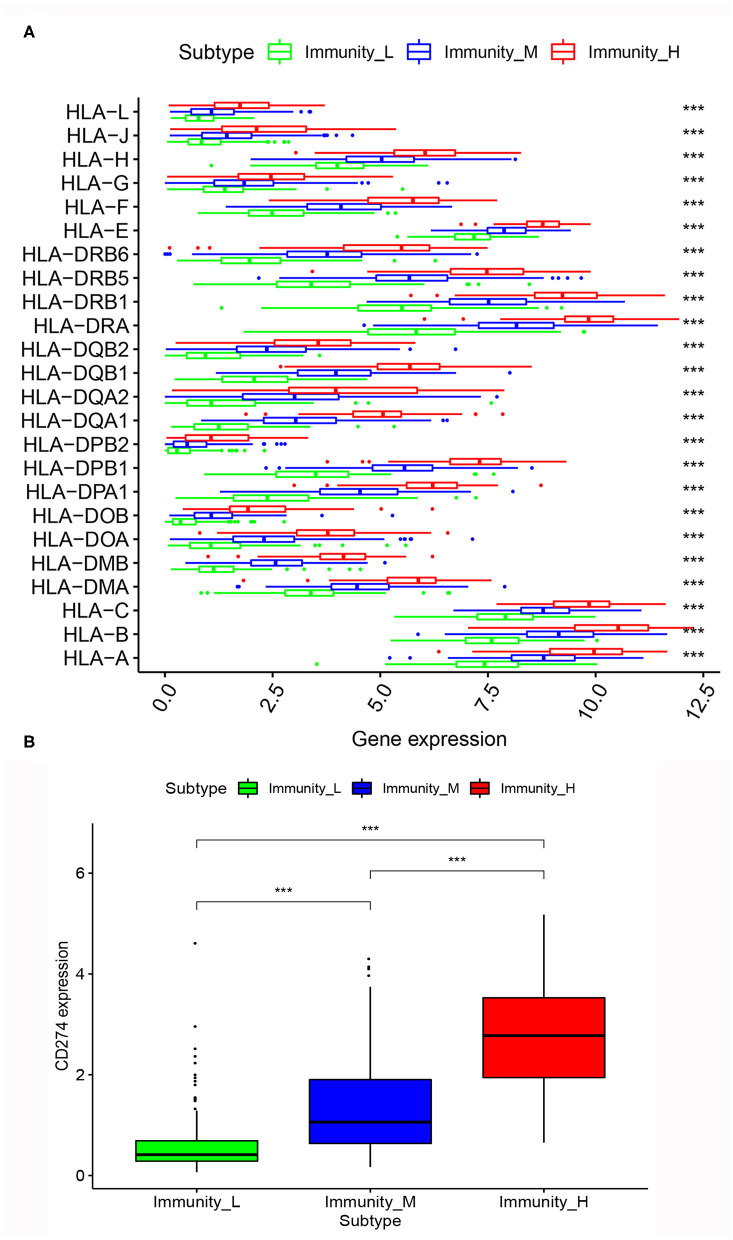
**(A)** Comparison of expression of multiple HLA genes among three MIBC subtypes. **(B)** Comparison of PD-L1 expression among three MIBC subtypes. *p < 0.05, **p < 0.01, ***p < 0.001.

After the differential analysis of HLA genes expression among the three MIBC subtypes, we found that, in the expression levels of HLA genes, the rank was Immunity_High > Immunity_Medium > Immunity_Low, consistently. The differences between the three subtypes were very significant (*p* < 0.001; Figure 5A).

Similarly, we calculated the expression level of PD-L1 among the three MIBC subtypes. The results also showed that Immunity_High had the highest PD-L1 expression levels, Immunity_Medium had the medium PD-L1 expression levels, and Immunity_Low had the lowest PD-L1 expression levels (*p* < 0.001; Figure 5B).

### Immunogenomic Subtypes in the Independent Validation Cohort

**Figure 6:**
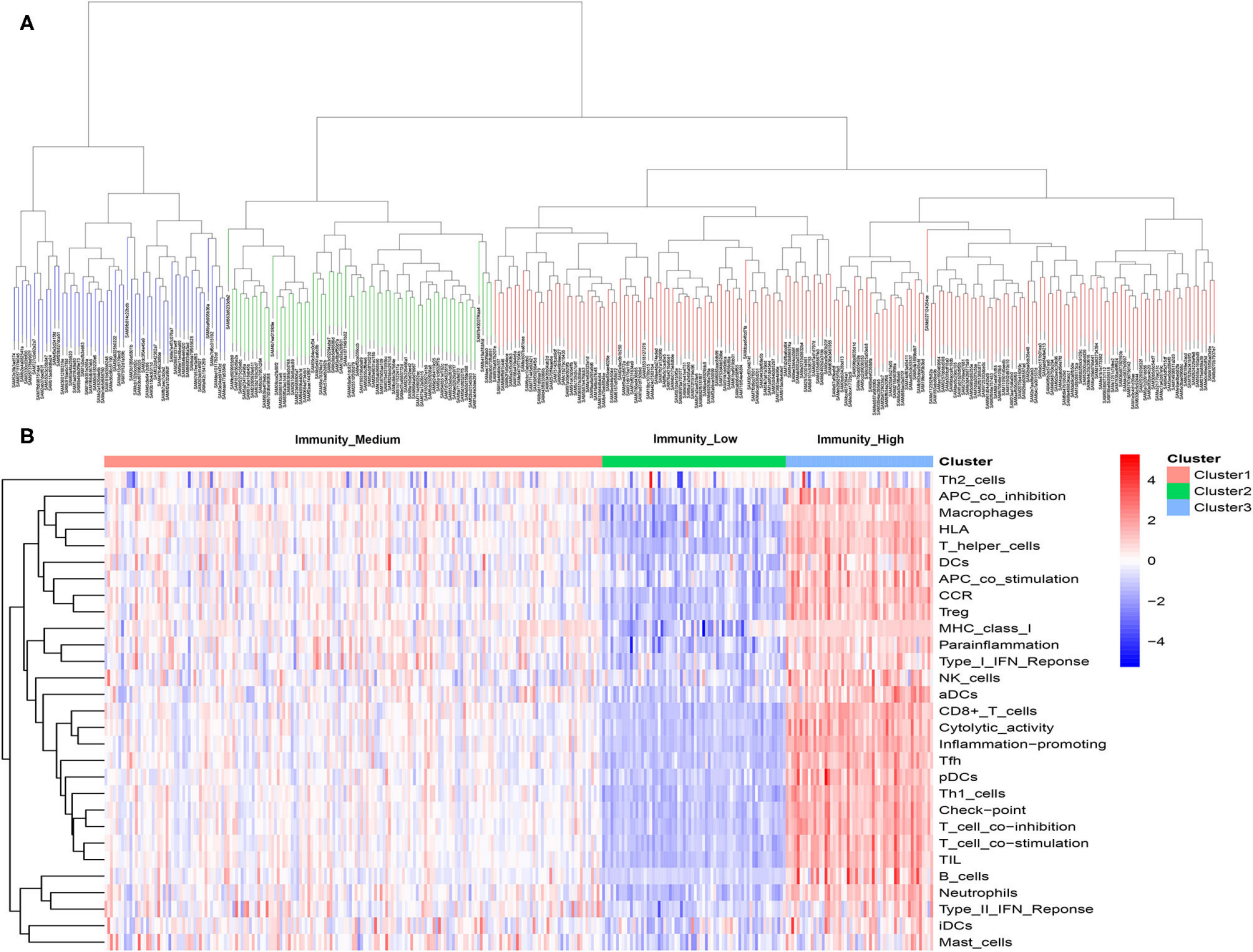
**(A)** Unsupervised hierarchical clustering of 298 muscle-invasive bladder cancer in the validation cohort. **(B)** Enrichment of 29 immune gene sets in the three clusters yielded by the hierarchical clustering. We named the three clusters Immunity_High, Immunity_Medium, and Immunity_Low.

Same as previous analyses in the discovery cohort, 298 patients were successfully divided into three clusters by hierarchical clustering. Similarly, the heatmap of expression levels of the 29 immune-associated gene sets show the obvious differences of the three clusters, we defined the three clusters as three MIBC immunogenomic subtypes named Immunity_High, Immunity_Medium, and Immunity_Low like before (Figure 6). The specific score and immune immunogenomic subtype of each sample were shown in Supplementary Table 5.

**Figure 7:**
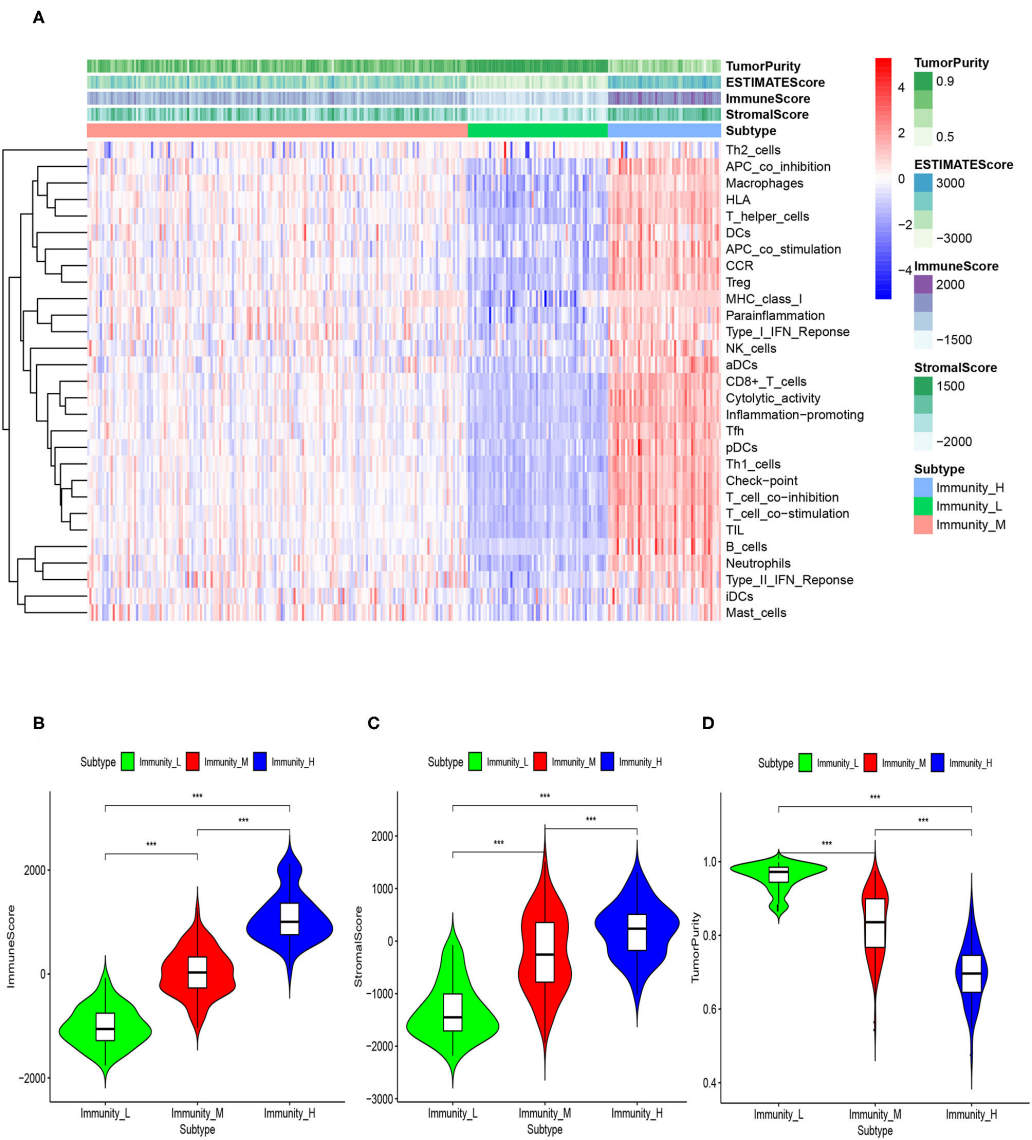
The correlation between immunogenomic classification of the validation cohort and the level of tumor immune infiltration was showed based immune score, stromal score, tumor purity score **(A)**. The violin plots specifically showed the differences in the three subtypes in terms of immune cell infiltration level **(B)**, stromal cell content **(C)**, and tumor purity **(D)**. *p < 0.05, **p < 0.01, ***p < 0.001.

Through the ESTIMATE algorithm, we have calculated the score of immune cell infiltration, stromal content, and tumor purity for three immunogenomic subtypes, respectively. As in the discovery cohort, Immunity_High had the highest score of immune cell infiltration and stromal content, while Immunity_Low had the highest tumor purity (*p* < 0.001). This showed that the immunogenomic subtypes applied in the independent validation cohort can also reflect the tumor immune microenvironment of patients to a certain extent (Figure 7).

### Association Between the Immunogenomic Subtypes and Immunotherapeutic Responsiveness

**Figure 8:**
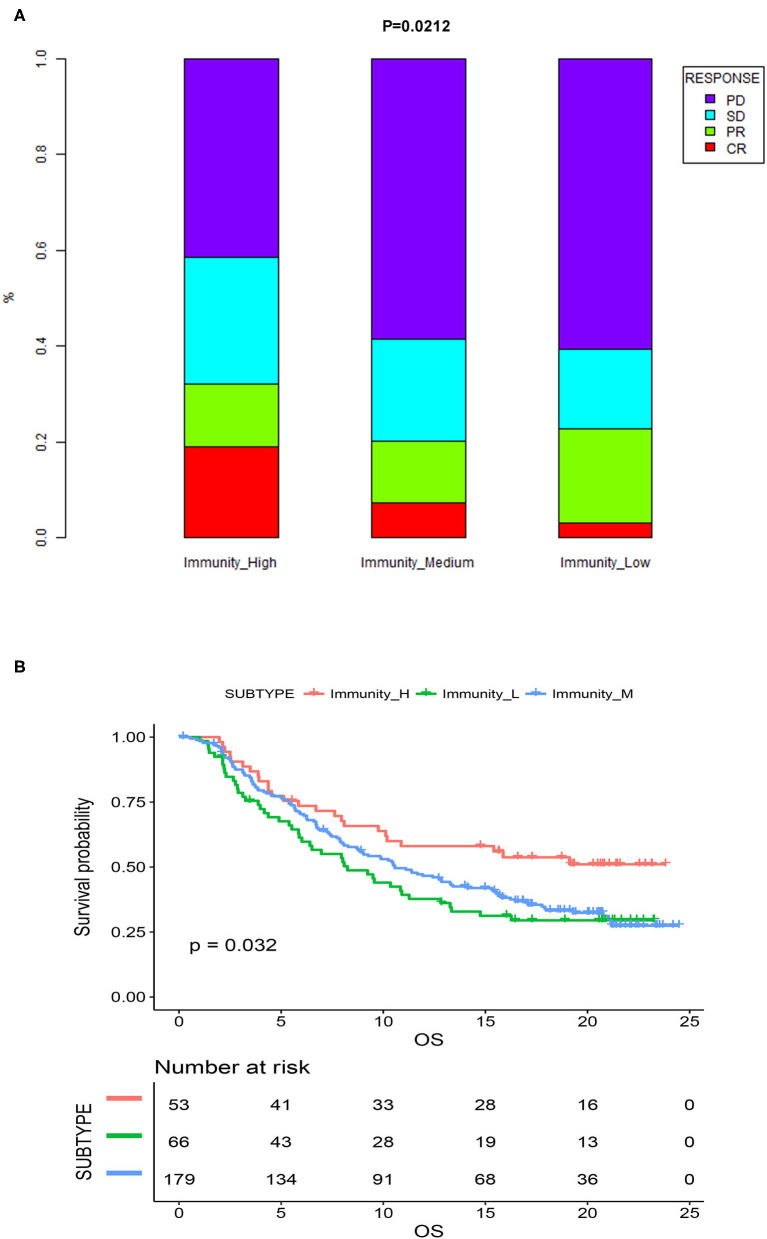
**(A)** Proportion of the four types of response to immunotherapy among different immunogenomic subtypes. **(B)** Kaplan-meier survival curves of MIBC patients treated with immunotherapy. CR, complete response; PR, partial response; SD, stable disease; PD, progressive disease.

Previous analyses demonstrated that immunogenomic subtypes were closely related to the immune microenvironment and prognosis of MIBC patients. We subsequently tested the association between the immunogenomic subtypes and immunotherapeutic responsiveness in the independent validation cohort with follow-up information on immunotherapy. The results showed that different immunogenomic subtypes had significant differences in immune response to immunotherapy (*p* = 0.021; Figure 8A) In patients of Immunity_High, 18.8% reached CR, while in patients of Immunity_Medium, and Immunity_Low, only 7.2, and 3% were able to reach CR. And 32% of patients of Immunity_High had a response to immunotherapy, which was also the most among three subtypes. Subsequent survival analysis results also showed that among all patients who were treated with immunotherapy, patients of Immunity_High had significantly better OS (*p* = 0.032; Figure 8B). Therefore, the immunogenomic subtypes we identified had the potential clinical value to predict the response and effect of immunotherapy.

## Discussion

In our study, we used ssGSEA technology to calculate the relative abundance of 29 immune cells in the discovery cohort of 399 MIBC samples. Subsequently, using unsupervised clustering, we can clearly identify three MIBC subtypes based on the ssGSEA scores: Immunity_Low subtype, Immunity_Medium subtype, and Immunity_High subtype. The results of survival analysis showed that Immunity_High subtype was associated with a significantly better prognosis among the three subtypes. We furtherly confirmed with a series of analyses that there was significant immune heterogeneity among the subtypes and Immunity_High subtype showed stronger immune activity in several aspects. In the validation cohort of 298 MIBC patients treated with immunotherapy from the IMvigor210, we validated the robustness of immunogenomic subtypes and the ability to predict the response to immunotherapy.

Researchers have identified some gene biomarkers associated with cancer immunotherapeutic responsiveness such as PD-L1 expression, tumor mutational burden (TMB), and deficient DNA mismatch repair (26–28). But the robustness of these biomarkers to predicting the efficacy of immunotherapy remains controversial. For example, although a clinical trial of BC has reported the median TMB of responders was two times higher than that of non-responders, there are still many responders with low TMB and non-responders with high TMB (9, 29).

There have been a series of studies that identified various subtypes for MIBC based on genomic profiling. These MIBC subtypes have differences in prognosis and biological characteristics, and show clinical significance in many aspects (30–33). In 2017, researchers analyzed the clinical pathological information and RNA-seq data of 412 bladder cancer patients in TCGA, and divided bladder cancer into 5 molecular subtypes (TCGA subtypes 2017), called Luminal papillary, Luminal infiltrated, Basal squamous, and neuronal subtypes. This study provided some new references for treatment options for bladder cancer. For example, the researchers reported that Luminal infiltrated subtype has a relatively higher lymphocyte infiltration and PD-1/PD-L1 expression, which indicates a higher probability of response to immunotherapy. In fact, this study focused on the differences in epithelial-mesenchymal transition status, carcinoma-*in-situ* scores, histologic features, and survival (30). Another group of researchers explored the characteristics of immune genes related to four distinct molecular subtypes (TCGA subtypes 2014) in MIBC published in 2014 by The Cancer Genome Atlas Network (TCGA) bladder analysis working group (31). The four subtypes are the basis of the TCGA subtypes 2017 mentioned above (32). They studied the immune characteristics of four TCGA subtypes by analyzing the immune, IFN-γ, IFN-α, and cytotoxic genes. They revealed an increased expression of immune-associated genes in Cluster IV and underactive immune environment in Cluster I, which may help increase the applicability of TCGA subtypes in immunotherapy options (31). But the two kinds of TCGA MIBC subtypes did not focus on more comprehensive immune-related genes or gene sets besides the tumor infiltration lymphocytes and immune checkpoints, as well as lacked follow-up data on immunotherapy. Therefore, MIBC subtypes based on more comprehensive immunogenomics characteristics and with immunotherapy follow-up data are still needed.

Another pan-cancer study based on TCGA data identified 6 immune subtypes for 33 non-hematologic tumors (33). The clustering of immune subtypes was based on the main 5 core immunogenomics characteristics including macrophages/monocytes, overall lymphocyte infiltration, TGF-β response, IFN-γ response, and wound healing. The author subsequently reported the differences of the immune characteristics and prognosis among the six immune subtypes, including differences in macrophage or lymphocyte signatures, Th1:Th2 cell ratio, extent of intratumoral heterogeneity, aneuploidy, extent of neoantigen load, overall cell proliferation, expression of immunomodulatory genes, and prognosis. This study comprehensively studied the immunogenomics characteristics of solid tumors and provided important knowledge in this field. And this study confirmed the feasibility of using immunogenomics features for tumor molecular typing. Similar to this study, the immunogenomic subtypes we obtained were also clustering according to a series of specific immunogenomics characteristics. Meanwhile, our immune subtypes also showed significant differences in multiple immune-related features. Our research focused on MIBC, and furtherly used actual clinical immunotherapy follow-up information to verify the predictive value of the immune response of subtypes, which is the strongness of our research compared with the pan-cancer study.

Through the calculation of ESTIMATE algorithm, we demonstrated that Immunity_High subtype had a significantly higher level of immune cell infiltration, stromal content, and lower tumor purity over Immunity_Medium and Imunity_L subtypes. In fact, researchers had demonstrated that immune cell infiltration levels and tumor purity are inversely proportional, and higher immune cell infiltration levels in multiple tumors that were positively correlated with the responsiveness to ICIs (34–36). Additionally, several studies on BC reported that BC could be divided into “hot” (enriched in immune cells infiltration) and “cold” (lack of immune cells infiltration) subtypes as a strong indicator for immunotherapy response (14, 15). Therefore, the obvious heterogeneity of our immunogenomic classification in terms of immune cell infiltration supports its potential clinical value in predicting immunotherapy responses.

After the correlation between the level of immune cell infiltration and patients' prognosis and treatment responsiveness had been confirmed by more and more studies, one emerging question has attracted attention. Was the proportion of immune infiltration cell types different in tumor samples with different prognosis and immunophenotype? Advances in computational methods had allowed us to take advantage of integrating genomic profiles and the state-of-the-art deconvolution accurately analyzed the relative proportions of various immune infiltration cell subsets, such as the CIBORSORT algorithm used in this study. CIBERSORT technology had been applied in a variety of oncology studies such as lung cancer and breast cancer, with an accuracy not less than flow cytology and immunohistochemistry (36–38). When we used CIBERSORT to calculate the proportions of 22 immune cell subsets in MIBC, we found that the three subtypes with different levels of immune cell infiltration also had significant differences in the proportion of immune infiltration cell types. For example, the proportions of activated CD4^+^ T memory cells, resting dendritic cells and CD8^+^ T cells were the highest in Immunity_High. The basic function of ICIs was to exert antitumor effects by enhancing the function of CD8^+^ T cells, while CD4^+^ T cells and dendritic cells could also play a supporting role in this process (16, 17). Previous studies on bladder cancer, prostate cancer, renal cancer, and colorectal cancer had reported that the level of CD8^+^ T cell infiltration was positively correlated with tumor prognosis and responsiveness to immunotherapy (39–41). Therefore, Immunity_High subtype, which was rich in CD8^+^ T cells, CD4^+^ T cells, and dendritic cells, had a higher possibility of immune response with an advantage in survival. We furtherly found that the ratio of CD8^+^/CD4^+^ and the ratio of CD8^+^/Treg were significantly higher in Immunity_High subtype than those in Immunity_Low subtype. Previous studies on breast cancer reported that lower ratio of CD8^+^/CD4^+^ is associated with poor prognosis, which is consistent with our findings (42). Another study believed the balance between the regulatory cells and effector cells of the body's immune system is essential to maintain an adequate immune response to fight diseases (such as cancer) and at the same time prevent damage to healthy tissues. This balance is caused by effector cells (such as CD8^+^ T cells) and regulatory T cells (or Tregs) maintain a mutual regulatory relationship (43). And consistent with our findings, a low CD8^+^/Treg ratio is associated with a poor prognosis.

Meanwhile, we observed that the proportion of macrophage M1 in Imunity H subtype was significantly higher than that in Imunity L subtype, while the proportion of macrophage M0 significantly decreased. The ratio of M1/M2 was also higher in Immunity_High subtype compared with it in Immunity_Low subtype. Tumor-associated macrophages (TAMs) were important components of the tumor immune microenvironment, including two functional states of M1 (anti-tumor) and M2 (tumor-promoting), and macrophage M0 could be transformed to M1 or M2. Previous studies have shown that the level of polarization of M0 to M1 might be associated with improved prognosis of the tumor, which was consistent with our finding that the prognosis of Immunity_High subtype with significantly increased M1/M0 was better (38, 44, 45).

Besides, we also found that Immunity_High subtype very significantly expressed more HLA genes, which indicted stronger immunogenicity and possible stronger immunotherapeutic responsiveness compared to the other subtypes. The HLA genes encode MHC I and MHC II molecules, and both Class I and Class II molecules present pathogen-derived short peptides to T cells to initiate an adaptive immune response. A previous study on metastatic melanoma suggested that HLA expression may affect the response of ICIs. The results showed that high tumor-specific MHC-I expression in response to anti-CTLA-4 therapy was essential, but anti-PD-1 or immune combination therapy was not effective. Meanwhile, tumor-specific MHC-II expression in response to anti-PD-1 therapy was essential, but it is not effective with anti-CTLA-4 or combined immunotherapy (43). Another study involving more than 1,535 cancer patients treated with ICIs found that the presence of more diverse HLA-I molecules was associated with increased survival after receiving immunotherapy. The authors believed that this might be due to the fact that such MHC molecules could bind more diverse peptides and provide a wider range of tumor antigens to T cells (46, 47).

In the Immunity_High subtype, the expression level of PD-L1 was also the highest among the three groups. The positive correlation between PD-L1 expression and immunotherapy response had been widely reported in many tumors including bladder cancer (8, 18, 19). Combining the above analysis, we could find that Immunity_High had very strong immune activity considering the level of immune infiltration, proportion of specific TILs, HLA richness and expression level, and PD-L1 expression level, and had relatively better clinical outcomes. MIBC patients of Immunity_High subtype were more likely to respond to immunotherapy, especially to anti-PD-1 / PD-L1 treatment.

There was an interesting phenomenon in the survival analysis of the discovery cohort, which the difference of OS between Immunity_medium and Immunity_low was not so significant as the difference of their immune characteristics. In a previous study on triple-negative breast cancer, the researchers also achieved similar results, that patients in Immunity_H had the best prognosis, but there was no significant difference in OS between Immunity_M and Immunity_L (23). We speculated that there may be the following reasons. Although our study found that patients in Immunity_M subtypes had significantly higher immune characteristics such as immune cell infiltration levels than those in immunity_L, the degree of this kind of advantages may not be sufficient to bring about significant improvement in survival. It may require sufficient immune cell infiltration and activation of immune-related pathways to reshape the balance between tumor immune surveillance and immune escape. At the same time, the identification of Immunity_High group with significantly better prognosis and immune response was more clinically meaningful and may help to screen out patients suitable for immunotherapy.

Our research identified a new MIBC classification method with potential clinical value in assessing immunotherapeutic responsiveness and prognosis. But we still had some limitations. First, our data was retrospective from TCGA. Although we had used a series of analyses to prove that this classification was distinguishable and clinically significant in many aspects, prospective clinical studies were still needed for further research. Second, although we had studied the potential value of this classification in distinguishing immunotherapeutic responsiveness in four aspects: tumor immunity infiltration, proportion of specific TILs, HLA expression, and PD-L1 expression, real-world data on immunotherapy of the discovery cohort was still missing. But our research demonstrated the feasibility and significance of this classification method, and pointed out the direction for further clinical studies.

## Conclusions

We identified a new MIBC classification based on transcriptome differences of 29 immune signatures in tumor samples from patients with MIBC. This classification had potential clinical implications for predicting prognosis and immunotherapeutic responsiveness.

## Data Availability Statement

Publicly available datasets were analyzed in this study. This data can be found here: TCGA data portal (https://portal.gdc.cancer.gov/).

## Ethics Statement

Ethical review and approval was not required for the study on human participants in accordance with the local legislation and institutional requirements. Written informed consent for participation was not required for this study in accordance with the national legislation and the institutional requirements.

## Author Contributions

QW and LY designed the study. XZho, SQ, and LN partly designed the study, performed the statistical analysis, and drafted the manuscript. DJ and KJ partly performed the statistical analysis. XZhe partly performed the data collection. All authors reviewed and final approval of the manuscript.

## Conflict of Interest

The authors declare that the research was conducted in the absence of any commercial or financial relationships that could be construed as a potential conflict of interest.

## Abbreviations

MIBC: muscle-invasive bladder cancer
ssGSEA: single-sample gene-set enrichment analysis
OS: overall survival
PD-1: programmed cell death 1 receptor
PD-L1: PD-ligand-1
CTLA-4: cytotoxic T lymphocyte-associated protein 4
ICIs: immune checkpoint inhibitors
TILs: tumor-infiltration lymphocytes
HLA: human leukocyte antigen
TAMs: Tumor-associated macrophages
CIBERSORT: Cell type Identification By Estimating Relative Subsets Of RNA Transcripts
ESTIMATE: Estimation of STromal and Immune cells in MAlignant Tumor tissues using Expression data.
