# Supplementary material for: Classification of Muscle-Invasive Bladder Cancer Based on Immunogenomic Profiling
Source: Front Oncol. 2020 Aug 18;10:1429. doi: 10.3389/fonc.2020.01429 (PMC7461944; doi:10.3389/fonc.2020.01429)
Supplement: Supplementary file 1 [file Table_1.DOCX]

Supplementary Table 1 – Descriptive characteristics of 339 patients in the discovery cohort from the The Cancer Genome Atlas Urothelial Bladder Carcinoma (TCGA-BLCA).

|  | Discovery cohort  (N = 399) |
| --- | --- |
| **Age, yr mean** ± **SD** | 67.9 ± 10.5 |
| **Gender, n (%)** |  |
| Female | 105 (26.3%) |
| Male | 294 (73.7%) |
| **Grade, n (%)** |  |
| Low grade | 20 (5.0%) |
| High grade | 376 (94.2%) |
| Unknown | 3 (0.8%) |
| **T stage, n (%)** |  |
| T2 | 117 (29.3%) |
| T3 | 225 (56.4%) |
| T4 | 57 (14.3%) |
| **Regional lymphonode invasion, n (%)** |  |
| No | 233 (58.4%) |
| Yes | 126 (31.6%) |
| Unknown | 40 (10.0%) |
| **Distant metastasis, n (%)** |  |
| No | 192 (48.1%) |
| Yes | 11 (2.8%) |
| Unknown | 196 (49.1%) |

Abbreviations: SD = Standard Deviation,
