# Supplementary material for: Classification of Muscle-Invasive Bladder Cancer Based on Immunogenomic Profiling
Source: Front Oncol. 2020 Aug 18;10:1429. doi: 10.3389/fonc.2020.01429 (PMC7461944; doi:10.3389/fonc.2020.01429)
Supplement: Supplementary file 2 [file Table_2.DOCX]

Supplementary Table 2 – Descriptive characteristics of 298 patients in the validation cohort from study IMvigor210

|  | Validation cohort  (N = 298) |
| --- | --- |
| **Gender, n (%)** |  |
| Female | 65 (21.8%) |
| Male | 233 (78.2%) |
| **Race, n (%)** |  |
| White | 270 (90.6%) |
| Black or African American | 9 (3.0%) |
| Asian | 7 (2.3%) |
| Other or unknown | 12 (4.0%) |
| **Baseline ECOG score n (%)** |  |
| 0 | 121 (40.6%) |
| 1 | 165 (55.4%) |
| 2 | 12 (4.0%) |
| 3 |  |
| **Response to immunotherapy** |  |
| CR | 25 (8.4%) |
| PR | 43 (14.4%) |
| SD | 63 (21.1%) |
| PD | 167 (56.0%) |

Abbreviations: ECOG = Eastern Cooperative Oncology Group, CR = Complete Response, PR = Partial Response, SD = Stable Disease, PD = Progressive Disease
